# Glucose-6-phosphate dehydrogenase (G6PD) mutations and haemoglobinuria syndrome in the Vietnamese population

**DOI:** 10.1186/1475-2875-8-152

**Published:** 2009-07-10

**Authors:** Nguyen Thi Hue, Jean Paul Charlieu, Tran Thi Hong Chau, Nick Day, Jeremy J Farrar, Tran Tinh Hien, Sarah J Dunstan

**Affiliations:** 1 Oxford University Clinical Research Unit, Hospital for Tropical Diseases, 190 Ben Ham Tu, District 5, Ho Chi Minh City, Vietnam; 2 Hospital for Tropical Diseases, 190 Ben Ham Tu, District 5, Ho Chi Minh City, Vietnam; 3 Centre for Tropical Medicine, Nuffield Department of Clinical Medicine, Oxford University, Oxford, UK; 4 Mahidol-Oxford Tropical Medicine Programme, Faculty of Tropical Medicine, Mahidol University, Bangkok, Thailand

## Abstract

**Background:**

In Vietnam the blackwater fever syndrome (BWF) has been associated with malaria infection, quinine ingestion and G6PD deficiency. The G6PD variants within the Vietnamese Kinh contributing to the disease risk in this population, and more generally to haemoglobinuria, are currently unknown.

**Method:**

Eighty-two haemoglobinuria patients and 524 healthy controls were screened for G6PD deficiency using either the methylene blue reduction test, the G-6-PDH kit or the micro-methaemoglobin reduction test. The G6PD gene variants were screened using SSCP combined with DNA sequencing in 82 patients with haemoglobinuria, and in 59 healthy controls found to be G6PD deficient.

**Results:**

This study confirmed that G6PD deficiency is strongly associated with haemoglobinuria (OR = 15, 95% CI [7.7 to 28.9], P < 0.0001). Six *G6PD *variants were identified in the Vietnamese population, of which two are novel (Vietnam1 [Glu^3^Lys] and Vietnam2 [Phe^66^Cys]). G6PD Viangchan [Val^291^Met], common throughout south-east Asia, accounted for 77% of the variants detected and was significantly associated with haemoglobinuria within G6PD-deficient ethnic Kinh Vietnamese (OR = 5.8 95% CI [114-55.4], P = 0.022).

**Conclusion:**

The primary frequency of several *G6PD *mutations, including novel mutations, in the Vietnamese Kinh population are reported and the contribution of *G6PD *mutations to the development of haemoglobinuria are investigated.

## Background

Deficiency of glucose-6-phosphate dehydrogenase (G6PD) is one of the most common enzymatic disorders of red blood cells in humans and has a varied clinical presentation [1]. The World Health Organization has defined the different G6PD variants according to the magnitude of the enzyme deficiency and the severity of haemolysis. The clinical expression of G6PD deficiency varies from severe enzyme deficiency to increased enzyme activity (class I to class V). The commonest clinical patterns are; 1) neonatal jaundice, 2) congenital haemolytic anaemia, 3) drug-induced haemolysis and 4) favism.

G6PD is the initial enzyme involved in the pentose phosphate pathway of erythrocyte metabolism. It is involved in the production of NADPH and indirectly of reduced glutathione necessary for the protection of the cells from oxidative stress. This enzyme is encoded by the *G6PD *gene, which is located at chromosome Xq28. The *G6PD *gene exhibits remarkable polymorphism in human populations and G6PD is known to have over 400 variants. These variants are distinguished by their electrophoresis and biochemical characteristics and some variants are not associated with significantly reduced enzyme activity in erythrocytes [2]. However, there are numerous mutations found in the *G6PD *gene, which are geographically isolated and cause a deficiency of the enzyme in erythrocytes [3-8]. Almost all G6PD deficiencies are caused by a point mutation of the genomic DNA resulting in an amino acid substitution [9]. To date, 140 mutations of the *G6PD *gene have been identified.

The incidence of G6PD deficiency is high in malaria endemic areas. There is evidence to support the hypothesis that G6PD deficiency confers a protective effect against illness following *Plasmodium *infection and genetic variability maintained at the G6PD locus appears to be an example of a balanced polymorphism [1]. In Africa and Southeast Asian countries where *Plasmodium falciparum *is endemic, the incidence of G6PD deficiency is estimated to be more than 10% (summarized by Matsuoka, 2005[10]). In contrast, in Japan, Northern China and Northern European countries, where malaria is historically not endemic, the incidence of G6PD deficiency is less than 0.1% (summarized by Matsuoka, 2005[10]). In the northern Vietnamese population, the incidence of G6PD deficiency is highly variable between ethnicities. The frequency of G6PD deficiency in the Kinh and the Mong ethnic groups, who traditionally have lived outside malaria transmission areas, is low (0.5% and 0.7%, respectively). The prevalence among ethnic groups living in the foothills of 4 provinces in the north of Vietnam (Thanh Hoa, Son La, Ha Giang, and Hoa Binh), the breeding area of the main malaria vector *Anopheles minimus*, ranges from 9.7% to 31% [11].

The blackwater fever (BWF) syndrome is characterized by severe intravascular haemolysis and anaemia producing dark urine in patients, and is also associated with malaria [12,13]. The pathogenesis of BWF remains unclear [14,15]. However, G6PD deficiency has been identified as a cause of haemolysis in patients receiving primaquine, or other oxidant drugs, and it is the single factor most often associated with acute intravascular haemolysis [3,4,8,16-18]. Chau *et al *showed in Vietnam that BWF was associated with quinine ingestion, malaria infection and G6PD deficiency, and that these three factors were not mutually independent and may interact [19]. The nature of the interaction is unclear, particularly as quinine is not an oxidant drug, like primaquine, known to cause haemolysis in G6PD deficiency. However, the overlapping risk factors suggest that haemoglobinuria caused by G6PD deficiency should not be regarded as a separate syndrome [19].

Though the prevalence of G6PD deficiency in Vietnamese populations has been reported, the genetic variants responsible for this deficiency and their associations with haemoglobinura have not been determined. G6PD mutations were screened in patients with haemoglobinuria and in G6PD deficient healthy individuals to identify the *G6PD *variants causing G6PD deficiency in the Vietnamese general population and to investigate the contribution of *G6PD *mutations to the development of haemoglobinuria.

## Methods

### Subjects

Three separate sample groups were collected from the Kinh and S'tieng ethnic groups at different times in Vietnam. Ethnicity was self-reported by the individuals enrolled in the study. The first group comprised of 266 healthy Vietnamese Kinh who lived in Ho Chi Minh City (HCMC; male N = 162, female N = 98, not recorded N = 4). In 2000, venous blood (5 ml) was collected in EDTA anticoagulant and G6PD deficiency was determined by the methylene blue reduction test [20]. From the remaining EDTA blood sample (3 ml) the plasma was removed and the cell pellet was stored at -20°C.

The second group comprised of 258 healthy Vietnamese S'tieng who lived in Binh Phuoc province. Finger prick blood samples were taken in 2001 and the level of G6PD was determined by the G-6-PDH kit (Sigma diagnostics, UK) according to the manufacturer's specification. The control samples used for this assay were healthy individuals with known G6PD deficiency. The remaining whole blood was stored at -20°C.

The third group was comprised of 82 patients with haemoglobinuria admitted to the Hospital for Tropical Diseases (HTD) HCMC between 1993 and 1996 [19] (male N = 75, female N = 5, not recorded N = 2). Macroscopically haemoglobinuria was defined as patients who had passed red or black urine. Microscopically haemoglobinuria was defined as a range of 0.1–1.8 g/dl of free Hb in urine, or Hb in the urine detected by Combur 9 Test Strips (3+ to 4+; Roche Diagnostics). Venous blood (2 ml) was taken from these patients and the level of G6PD was determined by screening RBCs using the micro-methaemoglobin reduction test [21]. All patients were investigated for the presence of malaria by thick and thin peripheral blood smears. 21/80 patients had confirmed malaria by the ParaSight F test, that detects *P. falciparum *HRP2. 15 of these 21 patients also had positive smears for P. *falciparum *and one was positive for both *P. falciparum *and *P. vivax *on admission to HTD. 5/21 patients had a positive blood smear result from the referring hospital but were negative on admission at HTD. *Plasmodium falciparum *trophozoites per microlitre of blood ranged from 20 to 75,360 (mean: 17615 per/μl). Transmission of malaria at the time of this study in this area of southern Vietnam occurs sporadically. The malaria patients in this study could therefore be defined as non-immune or partially immune individuals. Patients were then divided into two groups; haemoglobinuric patients with G6PD deficiency and haemoglobinuric patients without G6PD deficiency. 48/82 haemoglobinuric patients were defined as G6PD deficient based on a methaemoglobin reduction test of <5 at hospital admission (number of tests performed at admission N = 79) and discharge [number of tests performed at discharge N = 68; discharge at <1 week (N = 24), 1–2 weeks (N = 37), >2 weeks (N = 7)]. 35/82 haemoglobinuric patients had a further methaemoglobin reduction test at follow up (follow up was at >54 days post hospital admission and ranged from 54–365 days). From the follow up test 23 were confirmed as G6PD deficient. Therefore in this study 48/82 haemoglobinuric patients are defined as "probable G6PD deficient" and 23/82 are defined as "definite G6PD deficient". Plasma was removed from the remaining blood sample and the blood cell pellet was stored at -20°C.

### DNA extraction

DNA was extracted from frozen blood cell pellets or whole blood by the Nucleon genomic DNA extraction Kit (Tepnel Life Sciences, United Kingdom).

### DNA fragment amplification

Primers were designed to PCR amplify all 13 exons of the *G6PD *gene (table 1). The complete exonic regions were generated in 14 fragments ranging between 150–400 bp in length. PCR was performed in a 25 μL reaction containing 25 μM of each specific primer, 0.2 mM of each dNTP, 1 – 2.5 mM MgCl_2_, approximately 100 ng of DNA and 1 unit of AmpliTaq polymerase (Applied Biosystems, USA). PCR cycling conditions were as follows; 95°C for 5 min, annealing temperature (see Table 1) for 1 min then 30 cycles of 72°C for 1 min, 95°C for 30 s, annealing temperature for 1 min, and then 72°C for 5 min once. PCR products were separated on a 2% agarose gel containing 1 μg/ml ethidium bromide and bands were visualized by ultraviolet illumination (for specific PCR product sizes see Table 1).

**Table 1:** The primers and annealing temperatures used for PCR to amplify and sequence the exons of the *G6PD *gene

**Exons**	**PCR product size (bp)**	**PCR anealing temperature (°C)**	**Primers**
1A	305	62	F 5'-CTTGAACCCGCGAACAGGCGA
			R 5'-TCTCGGGCACCTGCGCTGGA
1B	365	68	F 5'-GCAGAGCAGCGGCAGCGGGTAT
			R 5'-TATTTTACCGCCGCGCGGCGCA
2	241	65	F 5'-CTCAAGAAAGGGGCTAACTTCTCAA
			R 5'-GCACTTCCTGGCTTTTAAGATTGGG
3+4	352	60	F 5'-CAGGCCAACTTCTAACCACACACCT
			R 5'-CCGAAGCTGGCCATGCTGGG
5	295	65	F 5'-CTGTCTGTGTGTCTGTCTGTCC
			R 5'-GGCCAGCCTGGCAGGCGGGAAGG
6	264	58	F 5'-ACTCCCCGAAGAGGGGTTCAAGG
			R 5'-GAGGCTCCTGAGTACCACCC
7	234	65	F 5'-CAAGGTCAGTTCCTCCACCTTGCC
			R 5'-GAAGAGTAGCCCTGCAGGGTGACT
8	164	72	F 5'-GGAGCTAAGGCGAGCTCTGGC
			R 5'-GGCATGCTCCTGGGGACTGGG
9	253	72	F 5'-CAAGGAGCCCATTCTCTCCCTT
			R 5'-TGCCTTGCTGGGCCTCGAAGG
10	318	72	F 5'-CTGAGAGAGCTGGTGCTGAGG
			R 5'-AGGCCGCCCACCCTCCACACT
11	160	65	F 5'-GCAGGCAGTGGCATCAGCAAG
			R 5'-CCCCATAGCCCACAGGTATGCAGG
12+13A	368	72	F 5'-TGTGTGCCACCGGCCTCCCA
			R 5'-GGGAAGGAGGGTGGCCGTGG
13B	364	68	F 5'-GTGGGTGAACCCCCACAAGGTC
			R 5'-TGGCCCCACTCAGGAGTGAGAC
13C	377	68	F 5'-CCATTCGTCTGTCCCAGAGCTTA
			R 5'-TGGGACAAGGAAGTGGGTCCTCA

### Mutation detection by single-strand conformation polymorphism (SSCP)

**Figure 1:**
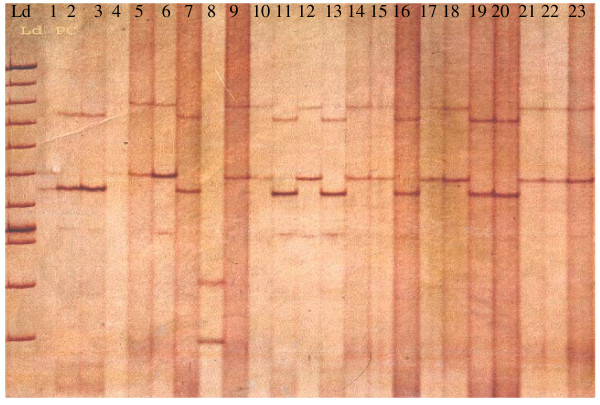
**Detection of G6PD variants by SSCP**. Different banding patterns, compared to the wild-type pattern, represent the presence of DNA sequence variation. Ld denotes 100 bp ladder. Lane 2 is the wild-type pattern.

PCR products were analysed by SSCP to detect mutations. Sequence variation can be recognized by band size changes on polyacrylamide gel (Figure 1), when comparing bands to a wild type control. PCR products were denatured at 80°C for 10 min prior to polyacrylamide gel electrophoresis. 5 μl of the denatured PCR product was mixed with loading buffer (98% formamide, 0.025% xylene cyanol FF and 0.025% bromophenol blue). The samples were separated on 6–8% polyacrylamide gels (acrylamide and bisacrylamide at a ratio of 99:1) in a tris borate buffer (TBE) containing 5% glycerol. If fragments were not separated effectively then the ratio of acrylamide to bisacrylamide was altered (39:1 or 19:1) and the percentage of glycerol was increased to 10%. Gels were electrophoresed in a vertical tank (Gibco BRL Sequencing System) at a constant power of 4W for 18 hours at 4°C. Gels were silver stained by standard procedures and then dried onto Whatman 3 MM paper using a vacuum gel drier.

### Cloning and DNA sequencing

Two or three samples for each unusual band pattern detected on an SSCP gel were prepared for sequencing to confirm the location of the sequence change. The PCR products were gel purified using a Geneclean kit (BIO 101, USA) and then cloned using the TOPO TA Cloning kit for Sequencing (Invitrogen, UK). Cloned plasmids for sequencing were extracted from *Escherichia coli *by the Perfectprep Plasmid Mini Kit (Eppendorf, Germany). PCR products that were sequenced directly where purified using the QIAquick PCR Purification kit (Qiagen, UK). Plasmid DNA templates were sequenced using the M13 forward and reverse primers, whereas PCR templates were sequenced using the specific primers that had initially generated the PCR fragment (Table 1). DNA sequencing was performed on a CEQ8000 capillary sequencer (Beckman, Singapore). The sequence of each sample was compared to the sequence of *G6PD *in GenBank (accession No. X55448) to identify the mutations.

### Statistical analysis

Fisher's exact test was used for all comparisons and was performed within the STATA software package.

## Results

Phenotypic screening for G6PD deficiency in healthy individuals from two ethnic groups, the Vietnamese Kinh and S'tieng, showed that the overall prevalence of the G6PD deficient phenotype in the southern Vietnamese population is relatively high at 11.3% (59/524). The difference in the prevalence of G6PD deficiency between the two Vietnamese ethnicities was not significant; 8.7% (23/266) in the Kinh and 14% (36/258) in the S'tieng (p = 0.07). Of the 23 G6PD deficient Kinh, 17 were male, five were female and one unknown (sex not recorded). Of the 36 G6PD deficient S'tieng, 28 were male and eight were female. In Vietnamese Kinh patients with haemoglobinuria, the frequency of "probable G6PD deficiency" was 58.5% (48/82). Twenty-eight percent (23/82) of haemoglobinuric patients had "definite G6PD deficiency." Of the 82 haemoglobinuric patients, five were female and 75 were male. All patients with probable G6PD deficiency were male (N = 48).

To identify the molecular cause of G6PD deficiency in this population the *G6PD *gene was screened for mutations in healthy individuals (of both Kinh and S'tieng ethnic groups) who were G6PD deficient, and in all haemoglobinuric patients (N = 82). Across the three groups eight polymorphisms were identified within the *G6PD *gene (Table 2). Five of these variants have been described in other populations, namely Gaohe Gaozhou (A95G; His^32^Arg), Coimbra "Shunde" (C11763T; Arg^198^Cys), Chinese-5 (C13184T; Leu^342^Phe), and Viangchan Jammu (G13031A; Val^291^Met) and nt13714C>T [9]. Three variants identified in this Vietnamese population are novel (two non-synonymous polymorphisms and one silent): Vietnam1 (G7A; Glu^3 ^Lys); Vietnam2 (T10148G; Phe^66 ^Cys); and Vietnam3 (C10170 T, Ser^73 ^Ser) (Table 2).

**Table 2:** *G6PD *variants present in the Vietnamese population.

				**Haemoglobinuria Kinh** **N = 82**		**Healthy Kinh** **N = 266**	**Healthy S'tieng** **N = 258**
				
**Variant**	**Variant name**	**Amino acid substitution**	**Mutation class^*a*^**	**G6PD+** **N = 34**	**G6PD-** **N = 48**	**G6PD-** **N = 23**	**G6PD-** **N = 36**
*Non-synonomous*							
G7A	Vietnam 1	Glu^3^Lys	*		1		
A95G	Gaohe Gaozhou	His^32^Arg	3		1		
T10148G	Vietnam 2	Phe^66^Cys	*			1	
C11763T	Coimbra Shunde	Arg^198^Cys	2	1		1	
G13031A	Viangchan Jammu	Val^291^Met	3,2	1	17	2	13
C13184T	Chinese-5	Leu^342^Phe	3	0	1	3	1
							
*synonomous*							
C10170T	Vietnam 3	Ser^73^Ser				1	
C13714T	nt13714C>T	Tyr^437^Tyr		10	14	9	21

The variant genomic positions are based on the GenBank sequence (accession no. genX55448X55448).

^*a *^class 1 = hereditary nonspherocytic haemolytic anaemia; class 2 = severe deficiency; class 3 = mild deficiency; class 4 = non-deficient; * unknown

In the healthy G6PD deficient group (both Kinh and Stieng) 37.3% (22/59) have no detectable mutation in the *G6PD *gene and 28.8% (17/59) have a synonymous polymorphism only. 33.9% (20/59) of the healthy G6PD deficient group have detectable non-synonymous *G6PD *variants. In the haemoglobinuria group, 25.6% (21/82) of patients harbour non-synonymous polymorphisms in the *G6PD *gene. Within the haemoglobinuria group of those patients with measured G6PD deficiency 39.6% (19/48) harboured non-synonymous *G6PD *polymorphisms, and 14/48 had a synonymous polymorphism (9/48 of these patients harboured both synonymous and non-synonymous *G6PD *polymorphisms). In contrast the patients without measured G6PD deficiency harboured significantly fewer non-synonymous *G6PD *polymorphisms (2/34 (6%), P = 0.001) but a similar number of synonymous polymorphisms (10/34 (29%), P = 1.0).

The most common polymorphism found in this study was the synonymous polymorphism nt13714C>T (C 13714 T; Tyr^437^Tyr). 42.1% (45/107) of G6PD deficient individuals in the Vietnamese population had this polymorphism and 29.3% (24/82) in the haemoglobinuria group. The second most common polymorphism found in this study was the Viangchan variant; 36% (13/36) of healthy G6PD deficient S'tieng, 9% (2/23) of healthy G6PD deficient Kinh and 22% (18/82) of Kinh with haemoglobinuria (35.4% with G6PD deficiency and 2.9% without).

Comparing the frequencies of G6PD deficiency in healthy Kinh and haemoglobinuric Kinh (table 3) demonstrates that G6PD deficiency is significantly associated with haemoglobinuria in the Kinh [comparison of "probable G6PD deficient" haemoglobinuric patients to healthy controls, OR = 14.9, 95%CI (7.7 to 28.9), P < 0.0001; comparison of "definite G6PD deficient" haemoglobinuric patients to healthy controls, OR = 4.11, 95%CI (2.04 to 8.34), P < 0.0001]. Some other possible reasons for haemoglobinuria other than G6PD deficiency in this patient population are malaria, anti-malarial treatment (quinine), other treatment, other infections (two patients had possible leptospirosis and two with possible sepsis) and thalassemia (one patient)".

**Table 3:** The frequency of G6PD deficiency and G6PD variants in patients with haemoglobinuria and healthy subjects

**G6PD**	**Hburia^*c *^Kinh** **N = 82**	**Healthy Kinh** **N = 266**	**Hburia Kinh G6PD-** **N = 48**	**Healthy Kinh G6PD-** **N = 23**	**OR**	**95%CI**	***P *value**
deficiency^*a*^	48	23			14.9	7.74 – 28.9	< 0.0001
definite^*b*^	23	23			4.11	2.04–8.24	< 0.0001
Viangchan Jammu			17	2	5.7	1.14 – 55.4	0.022
Chinese-5			1	3	0.14	0.002 – 1.9	0.09
Vietnam 1			1	0			
Gaohe Gaozhou			1	0			
Vietnam 2			0	1			

^*a *^deficiency includes definite and probable G6PD deficiency

^*b *^definite includes all cases with long term follow up that have confirmed G6PD deficiency

^*c *^haemoglobinuria

The frequency of the most common non-synonymous G6PD mutation (Viangchan) was significantly higher in G6PD deficient Kinh with haemoglobinuria than in G6PD-deficient healthy Kinh (OR = 5.7, 95%CI [1.14 to 55.4], P = 0.022). The Chinese-5 mutation has a low frequency in this population and the frequency was not significantly different between G6PD deficient Kinh with haemoglobinuria and G6PD deficient healthy Kinh (OR = 0.14, 95%CI [0.002 to 1.9], P = 0.09). Two variants, Vietnam1 and Gaohe Gaozhou, were only seen in the G6PD deficient Kinh with haemoglobinuria and not in the G6PD deficient healthy Kinh. In contrast, G6PD Vietnam2 was only seen in the G6PD deficient healthy Kinh group.

Table 4 summarizes the frequency of other factors that have previously been related to the development of haemoglobinuria and more specifically BWF. 58.5% of the patients with haemoglobinuria were G6PD deficient, 25.6% had malaria confirmed by a positive blood smear and 17% received anti-malarial treatment (either quinine or primaquine). The percentage of patients with haemoglobinuria that had non-synonymous mutations resulting in mild to severe G6PD deficiency was 25.6%.

**Table 4:** The frequency of factors that may contribute to the development of haemoglobinuria in the Vietnamese cohort

	**Haemoglobinuria Kinh N = 82**	**Healthy Kinh N = 266**	**Healthy S'tieng N = 258**
G6PD deficiency^*a*^	48 (58.5)	23 (8.65)	36 (13.95)
Suspected malaria^*b*^	34 (41.5)		
Confirmed malaria^*c*^	21(25.6)		
Received anti-malarials^*d*^	14 (17)		
Harbour G6PD polymorphism^*e*^	34 (41.5)	14 (0.05)	23 (0.09)
Harbour G6PD non-synonomous mutation	21(25.6)	6 (0.02)	14 (0.05)
Harbour G6PD published mutation	20 (24.4)	6 (0.02)	14 (0.05)

^*a *^deficiency includes definite and probable G6PD deficiency

^*b *^patients that are smear positive for malaria and patients that have been on anti-malarial treatment

^*c *^patients that are smear positive for malaria only

^*d *^patients that have received either quinine or primaquine anti-malarial treatment

^*e *^individuals that harbour 1 or more polymorphism

## Discussion

The frequency of G6PD deficiency in Southeast Asia is highly variable [22]. Within Vietnam the incidence of G6PD deficiency varies between the northern and southern areas and among ethnicities. In southern Vietnam, there is a high prevalence of G6PD deficiency in ethnic Kinh (8. 7%) and S'tieng (14%), who live in areas which are or were until recently highly endemic for malaria. In northern Vietnam, where malaria transmission is lower, G6PD deficiency is virtually absent [11].

Studies have shown that the distribution of G6PD variants also vary with geographical area and/or ethnic group. Matsuoka *et al *has reported that nine variants are present in China, nine in Malaysia, five in Thailand and four in Myanmar [23]. In Vietnam at least seven variants have been reported [23]. In this study, eight different polymorphisms were identified of which six resulted in an amino acid substitutions and three were novel mutations. The major G6PD variants reported in south-east Asian countries are G6PD Viangchan (G13031A) and G6PD Mahidol (G11658A). Almost all of the G6PD-deficient cases detected in Laos [22], Cambodia [10,24], Thailand [25,26] and Malaysian Malays [27] are G6PD Viangchan, while G6PD Mahidol is the most common variant in Myanmar [22,28,29]. G6PD Mahidol is also common in Thailand [25,30] and Malaysia [27].

In Vietnam, the G6PD Viangchan is the most common variant. A previous study in Lam Dong province also reported that G6PD Viangchan is the dominant G6PD variant in the Vietnamese population [23]. It can be hypothesized that the strong historical connection between Vietnam and China has resulted in the presence of common Chinese variants, such as Gaohe Gaozhou, Chinese-5 and Coimbra "Shunde" [31], in this Vietnamese population. Interestingly, the G6PD Mahidol mutation was not seen in this Vietnamese population. This is different from Thailand and Myanmar [29], where G6PD Mahidol is common. Notably, in this study, two novel non-synonymous variants (Vietnam1 and Vietnam2) and one novel silent polymorphism (Vietnam3) were identified. Even though the frequencies of these mutations are low, it is possible that a new generation of G6PD mutation is occurring in the Vietnamese population.

Studies in Southeast Asia report that G6PD Viangchan is in linkage disequilibrium with the silent polymorphism nt13714C>T [22,26,27]. In this study, 72.7% (24/33) of the subjects that harbour Viangchan also harbour nt13714C>T. The interpretation of these results could either be that the linkage disequilibrium between Viangchan and nt13714C>T is not absolute in the Vietnamese, or that detection of the nt13714C>T polymorphism by SSCP was not 100% sensitive. Findings to support the later are that the G6PD Viangchan mutation was demonstrated to be completely co-inherited with the silent polymorphism nt13714C>T in a previous study in the Vietnamese population [23]. Matsuoka *et al *[23] also found six previously published variants (Gaohe, Quing Yuan, Union, Canton, Kaiping and Bao Loc) in the Vietnamese Kinh of Lam Dong province. Of these, only the G6PD Gaohe variant was detected in this cohort of Vietnamese Kinh. In addition, 38% (41/107) of G6PD deficient people in this study had no identifiable mutation in the G6PD gene. Even though three novel G6PD variants were identified in this study, the SSCP method used may not have been a sensitive enough method to detect all mutations that exist in the population. In the future, direct sequencing of the G6PD gene would improve the sensitivity to detect G6PD variants. Alternatively some of these patients may not truly be G6PD deficient. There are two limitations of this study regarding the ascertainment of G6PD deficiency. Firstly, three different tests were used in the three subject groups, which were collected at different points in time, and in two different locations. This is not ideal, however in Vietnam, a rapidly developing nation, resources can vary over time. The use of two screening tests (methaemoglobin reduction test and methylene blue reduction test) may have lead to a reduction in sensitivity. Secondly, the specificity of the G6PD testing of haemoglobinuric patients may have been compromized, as the results depend on the time between haemolysis and the performance of the test. For the majority of haemoglobinuric patients screening for G6PD deficiency took place on hospital admission and at the time of discharge, so mis-classification could have occurred depending on the period between haemolysis and testing. A smaller subset of patients had further testing at follow up (>54 days post hospital admission), a time where G6PD testing would have been accurate. Therefore it could be a combination of the SSCP method lacking sensitivity and sub-optimal G6PD testing that is responsible for the lack of identifiable mutations in the G6PD gene in individuals deemed G6PD deficient in this study.

BWF has been associated with recent or active malaria, and with the ingestion of oxidant drugs such as primaquine. 25.6% of the patients with haemoglobinuria had confirmed malaria. In this Vietnamese Kinh cohort, the association between G6PD deficiency and haemoglobinuria was strong. The association between G6PD deficiency and haemolysis has been shown in at least four clinical syndromes, oxidative stress-induced haemolysis, favism, neonatal jaundice and chronic non-spherocytic haemolytic anaemia [26].

Of the G6PD variants, only G6PD Viangchan was shown to be associated with haemoglobinuria in the Kinh group. The G6PD Viangchan variant is one of the class 2 G6PD variants known to be associated with chronic haemolytic anaemia (according to Beutler, 1994 [32]). The frequency of the Chinese-5 variant, as well as other variants that result in a severe to mild enzyme deficiency, is low in the population, so comparisons are statistically unreliable. Currently, the rarity of haemoglobinuric patients from malaria endemic regions of Vietnam leaves one unable to expand these studies in a larger sample set.

The haemoglobinuric patients investigated in this study of G6PD variation were hospitalized between 1993 and 1996. Since the early 2000s it has been rare for the Hospital for Tropical Diseases in HCMC to admit patients with haemoglobinuria associated with malaria. This is may be related to a change in the treatment policy for malaria in Vietnam. In 1990 the first intervention trial investigating the use of artemisinin derivatives for the treatment of malaria in southern Vietnam was published [33]. Following the success of this and subsequent trials, artesunate replaced quinine in the national recommendations for the treatment of malaria in Viet Nam from 1998 onwards. After this date very little quinine has been used in Viet Nam. It is possibly this change in treatment for malaria from quinine to the artemisinin derivatives that has lead to significantly less drug induced haemolysis in Vietnamese individuals that are G6PD-deficient and infected with malaria.

## Conclusion

This study reports the primary frequency of several *G6PD *mutations in Vietnam. G6PD Viangchan, a common south-east Asian variant, was found to be the most prevalent and two novel non-synonymous variants (Vietnam1 and Vietnam2) and one silent polymorphism (Vietnam3) were identified in this Vietnamese population. G6PD deficiency is a major risk factor for haemoglobinuria in Vietnamese Kinh and within the G6PD deficient population G6PD Viangchan was significantly associated with haemoglobinuria.

## Abbreviations

G6PD: Glucose-6-phosphate dehydrogenase; BWF: Blackwater fever; SSCP: Single strand conformation polymorphism.

## Competing interests

The authors declare that they have no competing interests.

## Authors' contributions

NTH carried out the molecular genetics study, collected healthy samples, carried out the G6PD test using the G-6-PDH kit, performed the analysis and drafted the manuscript. JC participated in the design of the study. TTHC carried out the micro-methaemoglobin reduction test, recruited the patients and collected clinical data. TTH and ND were involved in recruiting the patients and collecting clinical data. JF conceived of the study, participated in its design and edited the manuscript. SJD participated in the data analysis, drafted and edited the manuscript
